# Resampling the pool of genotypic possibilities: an adaptive function of sexual reproduction

**DOI:** 10.1186/s12862-021-01850-5

**Published:** 2021-06-12

**Authors:** Donal A. Hickey, G. Brian Golding

**Affiliations:** 1 Department of Biology, Concordia University, 7141 Sherbrooke West, QC H4B 1R6 Montreal, Canada; 2 Department of Biology, McMaster University, 1280 Main St. West, ON L8S 4K1 Hamilton, Canada

**Keywords:** Sexual reproduction, standing genetic variation, Evolution of sex

## Abstract

**Background:**

Natural populations harbor significant levels of genetic variability. Because of this standing genetic variation, the number of possible genotypic combinations is many orders of magnitude greater than the population size. This means that any given population contains only a tiny fraction of all possible genotypic combinations.

**Results:**

We show that recombination allows a finite population to resample the genotype pool, i.e., the universe of all possible genotypic combinations. Recombination, in combination with natural selection, enables an evolving sexual population to replace existing genotypes with new, higher-fitness genotypic combinations that did not previously exist in the population. This process allows the sexual population to gradually increase its fitness far beyond the range of fitnesses in the initial population. In contrast to this, an asexual population is limited to selection among existing lower fitness genotypes.

**Conclusions:**

The results provide an explanation for the ubiquity of sexual reproduction in evolving natural populations, especially when natural selection is acting on the standing genetic variation.

## Background

The evolutionary persistence of sexual reproduction has been the subject of much debate among biologists [1, 2]. Here, we illustrate the effects of sexual reproduction in a finite population where selection acts on the standing genetic variation [3–9]. One of the earliest explanations for the function of sex was that it combines different beneficial mutations in a single individual, resulting in fitter genotypic combinations [10, 11]. Those authors focused on new beneficial mutations rather than on the standing genetic variation. Maynard Smith [12] pointed out that, given a low mutation rate, small populations might not contain multiple mutations at the same time and hence would not benefit from recombining such new advantageous mutations. By focusing on the standing genetic variation rather than only new mutations, we avoid Maynard Smith’s concern. Of course, new mutations will eventually contribute to the standing genetic variation once they reach significant frequencies.

Biologists are well aware that Mendelian genetics in an outbreeding sexual population is essentially a game of chance. In this study, we use a lottery analogy to explain, in simple terms, a major biological function of genetic recombination in a sexually reproducing population. We point out that in the case of inheritance, the lottery is biased by selectively induced, heritable changes in the frequencies of individual genetic variants. We argue that it is the interaction between random recombination and deterministic natural selection that allows sexual populations to evolve much more efficiently than asexual populations. This provides a consistent, short-term selective advantage for sexual reproduction relative to asexual reproduction.

The genetic lottery is special in a number of ways and by taking these special features into account we can better understand how recombination provides a fitness advantage relative to asexual reproduction. The first feature to be considered is that the winning combination, or optimal genotype, does not change drastically and unpredictably from one generation to the next. Environments do change, but this change usually happens gradually over many generations. Thus, the current environment is a reasonable estimator of environmental conditions in at least several following generations. The second point to consider is that the number of possible genotypic combinations can be many orders of magnitude larger than the total population size (see discussion below). This means that the chance that even a single individual within the entire population will have the optimal genotype is extremely remote. The third point is that both genetic lotteries and many conventional lotteries can have partial winners even in the absence of an overall jackpot winner. In both cases there is an advantage in having at least some of the winning numbers. Finally, the crucial difference between a conventional lottery and the genetic lottery is that inheritance provides an informational feedback each generation regarding the “winning” genotypic combination. We explain this point in more detail below.

Students of genetics are familiar with the process of generating various genotypic combinations of alleles at two, three or four variable loci. But we rarely stop to think how this plays out at the genomic level. Eukaryotic genomes are estimated to contain approximately 20,000 genetic loci and many of these loci are genetically polymorphic [13]. This discovery of abundant genetic variability in natural populations means that the impact of recombination is much greater than what was previously envisaged [14]. If we take the overly conservative estimate that only 1 % of the gene loci within the genome are genetically variable, we are still left with 200 genes. If we make a further conservative assumption that none of the genes has more than two allelic variants, we can calculate that there are 2^200^ possible genotypic combinations. This number is larger than 10^60^ which in turn is several orders of magnitude larger than the estimated number of atoms in the solar system. Obviously, no biological population is large enough to contain even a single individual representing each of these combinations. In fact, real populations, because of their finite size, contain only a tiny fraction of all possible genotypic combinations.

The relationship between the number of genetically variable genes and the total number of possible genotypic combinations was first noted by East [15]. He pointed out that ten genes with two alleles each could generate more than a thousand different genotypes and that twenty such genes could generate more than a million genotypic combinations. This calculation was subsequently considered by Iles et al. [16] indicating that the number of genotypes could be greater than the population size. We have previously investigated the implications of this exponentially increasing number of possible genotypes for the cost of sex [12] and the cost of selection [17]. We showed that although an asexual clone has an initial two-fold replication advantage, that excess relative fitness is eroded as the genotypic composition of the sexual population evolves in response to selection [18]. Subsequently, we focused on the cost of selection, also known as Haldane’s dilemma [19]. According to Haldane [17], the rate of increase in allele frequencies would be impeded by adding more loci to the selection process. We showed that this was not true for sexual populations; allele frequencies increased at the same rate regardless of the number of loci under selection. Here, we compare the average fitness of sexual and asexual populations in response to multi-locus selection. We also asked if it was necessary to invoke selection at hundreds of loci, as had been done in our previous studies. In order to answer this question, we used numerical simulations to compare the average fitness of sexual and asexual populations when only twenty loci were subject to selection.

## Results

**Fig. 1:**
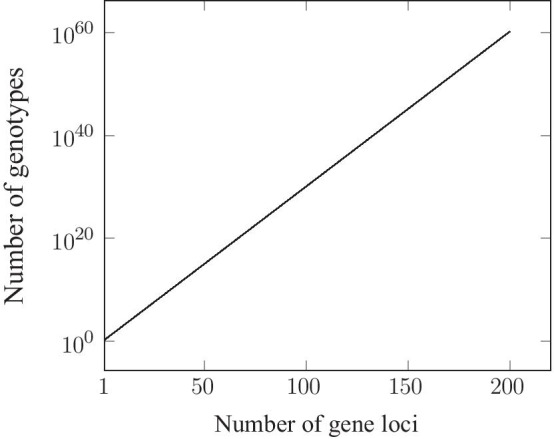
The relationship between the number of genetically variable genes and the number of possible genotypic combinations. The graph shows the results for up to 200 biallelic loci. The Y axis is on a log scale. The number of genotypic combinations is estimated by 2^ L^, where L is the number of loci

The theoretical relationship between the number of variable genes and the corresponding number of genotypic combinations is shown in Fig. 1. From the Figure it can be seen that once we reach more than thirty genetic loci, the number of possible genetic combinations already exceeds most real population sizes, especially those of macroscopic plants and animals. For example, 30 biallelic loci can generate more than one billion different genotypes. Once we reach more than 100 loci, the numbers of genotypic combinations become astronomically large (see Fig. 1).

In successive rounds of a conventional lottery, new tickets are printed using the same expected frequencies of numbers as in the previous round. But in the genetic lottery these frequencies are modified based on the genetic composition of the selected parents. For example, early in the process the frequency of favorable alleles at individual loci may have risen from 0.01 to 0.015 due to selection. In this case, and assuming 100 loci under selection, random recombination will generate new diploid genotypes with an average of 3 favorable alleles per diploid genome, a slight increase from the original value of 2. As the process continues, both the allele frequencies at individual loci and the total number of favorable alleles per chromosome will increase concomitantly until, eventually, all chromosomes will contain the maximum number of favorable alleles. In a conventional lottery, such a continual adjustment of the individual frequencies would be considered a blatant case of cheating. But it is precisely this “trick” that nature uses to arrive at a genotypic combination that appears to be mathematically unattainable at the outset. This process is like the multiplicative weights update algorithm, MWUA, that has been used as an optimisation method in game theory and machine learning [20, 21]. Specifically, random recombination updates the distribution of genotypes each generation based on the new, post-selection allelic frequencies.

**Fig. 2:**
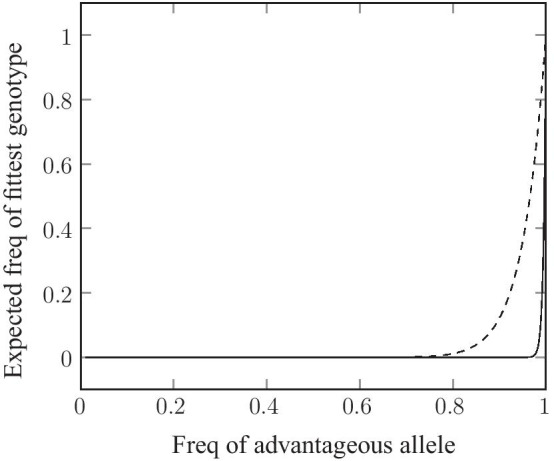
The relationship between the increase in the frequency of favorable alleles at individual genetic loci (X axis) and the expected frequency of the optimal genotypic combination. As can been seen from the figure, the frequency of the optimal genotype stays close to zero over most of the frequency range at the individual loci. This effect is most pronounced for 200 gene loci (solid line) but it can also be seen for as few as 20 loci (dashed line). The frequency of the optimal genotype, G, is estimated by G = p_1_.p_2_.p_3_……p_L_, where p represents the frequency of the favorable allele at individual loci and L is the number of loci

All genotypes in the population will have a relatively low fitness initially, but natural selection will tend to favor “the best of the bad lot”. These early rounds of selection will result in incremental increases in the frequency of favourable alleles at each of the 100 loci. Recombination will then generate new random combinations of these alleles. And since the individual allele frequencies have increased slightly, the expected frequency of genotypes with higher numbers of favorable alleles per chromosome will also increase automatically. The relationship between the rising allele frequencies at each locus and the frequency of the “jackpot combination” (that is homozygous for the favorable allele at all 100 loci) is shown in Fig. 2. As can be seen from the Figure, it is only toward the end of the process that the optimum genotype appears, where it then rises dramatically in frequency. This pattern can be explained mathematically by saying that the frequency of the optimum genotype is an extreme power function of the individual allele frequencies.

**Fig. 3:**
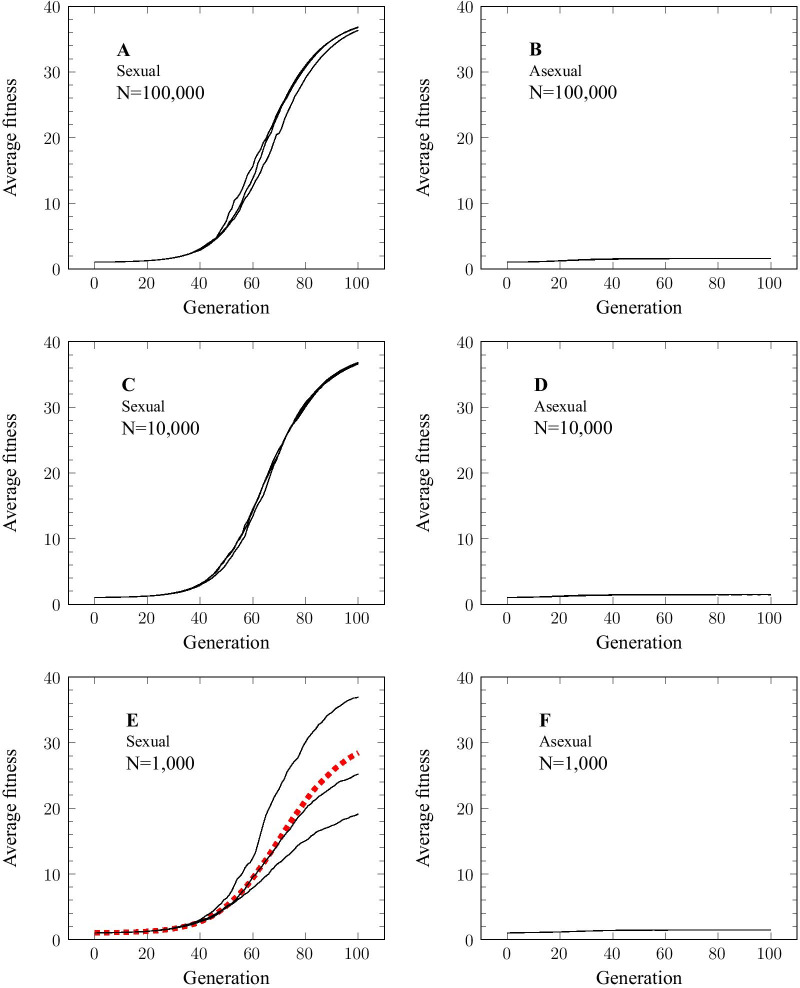
Fitness changes in sexual and asexual populations. Numerical simulations were performed using 20 unlinked loci. Selectively favored alleles had an initial frequency of 0.01. The selection coefficient for a favored allele was 0.1 and fitnesses were multiplicative between loci. Selection was implemented during the diploid phase of the life cycle. Three different population sizes were used: N = 100,000 (Panels **A** and **B**); N = 10,000 (Panels **C** and **D**); N = 1,000 (Panels **E** and **F**). Three replicates were performed for each simulation. Because of the greater variation between replicates in the case where N = 1,000, a further 30 replicates were performed for this case and the results are shown as a dashed line in Panel **E**

We performed numerical simulations in order to validate our theoretical calculations. As can be seen in Fig. 2, there is no need to assume hundreds of loci in order to see the effect of recombination: even if we consider as few as twenty loci, there is still a considerable effect (see dashed line in Fig. 2). Consequently, we performed the simulations using only twenty selected loci; the results are shown in Fig. 3. We used three different population sizes. From the Figure we see that there is a clear difference in fitness between the sexual and asexual populations at all three population sizes. In all three cases, and despite identical starting conditions, the fitness of the sexual population rises to a much higher level than that of the asexual population. It may seem surprising that the asexual population shows such a poor response to selection even when the population size is as large as 100,000 diploid individuals (see Fig. 3, Panel B). But given that the favorable alleles have an initial frequency of only 0.01, the expected frequency of diploid genotypes that contain 6 or more favorable alleles in the initial population is very low, of the order of 10^− 6^. Consistent with this expectation, the fittest diploid individuals in the initial populations (both sexual and asexual) had a total of only 5 or 6 favorable alleles. Selection among the asexual individuals led to the fixation of such genotypes. The sexual population, however, was not limited by the genotypic diversity present in the initial population. Instead, it could access fitter genotypic combinations though recombination as the allele frequencies rose in response to selection at the individual loci. Eventually, the sexual population contained individuals that were homozygous at all 20 loci, i.e., containing a total of 40 favorable alleles per individual.

The results were highly consistent between replicates for population sizes of 10,000 and 100,000 individuals (see Fig. 3, panels A and C), but there was more variability between replicates for the population of 1000 individuals (Fig. 3, Panel E). Closer inspection of the data revealed that this was due to the loss of allelic variation due to genetic drift in the early generations of the simulation. In one replicate the favorable allele was lost at 2 out of the twenty loci; it was lost at 3 loci in another replicate. Because of this variability between replicates, we performed 30 additional replicates of this particular simulation and the average results are shown as a dashed line in Fig. 3, Panel E. Despite this random loss of variability, however, the sexual population still far outperforms the asexual population (Compare Panels E and F in Fig. 3).

Overall, the simulations indicate that recombination provides a very significant advantage even in the case of a relatively modest number of selected loci (twenty in this case).

## Discussion

The exponentially increasing number of genotypic combinations as the number of genetically variable loci increases was first pointed out by East [15]. Subsequently, Iles et al. [16] used this relationship to point out that multi-locus genotypes could be very rare even in very large finite populations. As illustrated in Fig. 1, once the number of loci under selection exceeds twenty or thirty, the number of possible genotypic combinations becomes much greater than most biological population sizes. This means that natural selection cannot act on the full range of genotypes because, when many loci are under simultaneous natural selection, the optimum genotype is normally absent from the population. Thus, selection cannot act directly on this genotype, although we tend to think of the process in such teleological terms. In fact, the production of the optimal genotypic combination is simply the automatic consequence of recurrent rounds of natural selection and recombination. Most of the selection occurs among genotypes of varying degrees of intermediate fitness. Although the effects of recombination are purely random [22], yet the interaction between selection and random recombination provides an efficient mechanism for the eventual production of the optimal genotype. The power of recombination lies in the fact that it allows a finite population to effectively resample genotypes from a virtual, infinite population with the current allele frequencies [18, 23, 24]. It is in this way that it produces higher fitness genotypes that were not previously present in the population.

Previous studies of recombination in finite populations have focused on the adjustment of existing genotype frequencies [16, 25, 26]. Here, our focus is not on the frequencies of existing genotypic combinations, but rather on the production of new genotypes that did not previously exist in the population. As fitter haplotypes increase in frequency, recombination can act to produce still fitter haplotypes [16]. Previously, adaptive evolution based on selection at many genetic loci was seen as potentially very costly in terms of selective deaths [17]. However, Haldane’s calculations were based on a model of selection among fixed genotypes. Once we include the possibility of recurrent disassembly and reassembly of genotypes through random recombination, the problem envisaged by Haldane disappears [19].

One of the earliest proposals for the advantage of sex was that it generates genetic variants [27]. While this is true, sexual reproduction does not usually increase the overall level of variation relative to that in the previous generation. Rather, it replenishes the genotypic variation that would have otherwise been eroded by selection and random genetic drift. Even more important, it continually shifts the genotypic distribution towards a greater average fitness, as we have shown above. Without sex and recombination, the adaptive evolutionary process would be greatly impeded. Some classic population genetics studies [17] envisaged selection as performing an exhaustive search among all possible genotypic combinations. But this is not necessary in a sexual population; instead, a more heuristic process occurs whereby the best available sub-optimal genotypes are selected and then recombined to produce some slightly higher fitness genotypes. Repeated cycles of this process lead to the eventual production of the optimum genotypic combination.

## Conclusions

Our main conclusion is that a major adaptive role of sexual reproduction lies in its ability to translate increasing allele frequencies at several individual loci into increasing numbers of favorable alleles per chromosome. In the short term, natural selection acts on whole genotypes but recombination then disassembles these selected genotypes and recombines them into new genotypic combinations, some of which have higher-fitness than any of the previously-existing genotypes. This two-step iterative process enables an outbreeding sexual population to harvest all of the favorable alleles that are present in the population, regardless of their initial genetic background, and to combine them into a single optimal genotype. Without recombination, the efficient production of such genotypes would be mathematically unattainable in a finite population.

## Methods

We assumed a finite population with discrete, non-overlapping generations. For computational simplicity, only biallelic loci were considered although the arguments are equally applicable to multiallelic loci. Fitness interactions between loci were assumed to be multiplicative, i.e., no epistatic interactions. The results do not, however, depend on the fact that the fitnesses are multiplicative; additive fitnesses would yield very similar results. Indeed, the only requirement is that the overall genotypic fitness be an increasing function of the number of favorable alleles.

Numerical simulations were performed using a strategy similar to that described in Hickey and Golding [18]. In this study, we assumed 20 unlinked loci that were initially in linkage equilibrium. Populations were initiated with a frequency of 0.01 for the favorable allele at each individual locus. Favorable alleles had a selective advantage of 0.1. There were no epistatic interactions between loci, i.e., fitness interactions were multiplicative. For the sexual populations, new recombinant genotypes were generated using the parental post-selection allele frequencies at each locus.

Separate simulations were carried out for populations containing various numbers of diploid individuals; N = 1000, N = 10,000 and N = 100,000. We did three replicates of each simulation. Since our main argument is based on the relationship between the population size and the possible number of genotypes, we wished to investigate the effects of varying the population size.

The raw data and the source code for the simulations are available at: https://github.com/gbgolding/Resampling-Genotypes.

## Data Availability

The raw data and the source code for the simulations are available at: https://github.com/gbgolding/Resampling-Genotypes.
